# Tibiotalocalcaneal Nail: A Novel Tool in the Management of Old Neglected Ankle Dislocation With Deformity

**DOI:** 10.7759/cureus.68746

**Published:** 2024-09-05

**Authors:** Gursimran Singh, Nareshkumar Dhaniwala, Anmol Suneja

**Affiliations:** 1 Department of Orthopedics, Datta Meghe Institute of Higher Education and Research, Wardha, IND

**Keywords:** ankle dislocation, ankle joint reconstruction, complex ankle injury, orthopedic management, tibiotalocalcaneal nail

## Abstract

Ankle dislocations, particularly those that are old and neglected, pose significant challenges in orthopedic management due to the development of arthritic changes and surgical difficulties of reduction. The optimal treatment often involves stabilization and realignment to ensure proper healing. The closed reduction is rarely achieved in old neglected dislocations. Open reduction with internal or external fixation is the treatment for old neglected dislocations. Ankle and subtalar arthrodesis ensure painless plantigrade foot. The case report describes a case of an 8-month-old unreduced ankle dislocation with equinus deformity managed with ankle and subtalar arthrodesis using tibiotalocalcaneal nail. The equinus deformity was corrected and painless stable ankle joint was achieved.

## Introduction

When high-velocity trauma occurs on the ankle, the bones of ankle joints shift from their original position, and an ankle dislocation occurs. The approximate incidence of ankle dislocation is 0.065% of the cases of all ankle injuries. Sporting and motor vehicle accidents are the most common causes of ankle dislocation. The injuries that cause inversion at the ankle joint lead to posteromedial displacement, and the eversion injuries cause lateral dislocations. Superior dislocation is caused when the everted foot is dorsiflexed. The signs and symptoms consist of agonizing pain, discoloration, visible deformity of the ankle, and marked puffiness. The displacement can be posterior, anterior, lateral, or medial and can also be associated with malleolar fractures [1]. Posteromedial dislocation is the most common type of ankle dislocation. The diagnosis is confirmed by clinical examination and imaging modalities such as plain radiographs that help identify the dislocation and any concomitant fractures. These cases are managed with open reduction and internal fixation (ORIF) or open reduction and external fixation under anesthesia, repair of torn ligaments, and postoperative immobilization for six to eight weeks, followed by rehabilitation [2]. External fixation is done during damage control, or local or systemic conditions that contraindicate internal fixation.

Neglected cases are cases of ankle dislocation that remain untreated for three weeks or more. Open reduction can be done in old, unreduced, and neglected cases, but it leads to painful joint movements due to damaged articular cartilage. Ankle and subtalar arthrodeses remain the procedure of choice in such cases. Arthrodesis is one of the alternatives given that conditions such as the patient's age/functionality, presence of malleolar fractures, or posterior process of the talus should be taken into account when opting for this management that involves fixing the ankle and hindfoot.

The TTC nail gives immense support to fractures or dislocations that are not solidly fixed through the traditional fixation methods. The nails used are expert hindfoot arthrodesis nail and T2 ankle arthrodesis nail depending on the availability and surgeon’s preference. In this case, a T2 ankle arthrodesis nail is used. The guide wire is inserted through the plantar aspect of the calcaneum and advanced to the center of the talar dome and tibia. This direction is confirmed in both anteroposterior and lateral views. A 2 cm incision is taken at the entry point of the guide wire, followed by serial reaming along the guide wire. After the insertion of the tibiotalocalcaneal (TTC) nail, proximal locking is done first, followed by locking through the dynamic position in the talus. In the final stage, the nail-mounted internal compression screw is tightened [3]. The method also reduces infections and soft tissue damage and improves stability for early weight bearing and functional recovery compared to external fixators, among others. The TTC nail, in general, is a useful instrument for treating severe and complicated ankle injuries, promoting efficient healing and functional recovery [4].

The complications associated with the use of TTC nails for ankle arthrodesis are infections, peri-implant fracture, nonunion/malunion of bones, and tissue irritation. Infection in these cases leads to implant failure and the need for revision surgery. The fracture of the talus and calcaneum is a known complication of TTC nails.

Isolated ankle dislocations are usually managed with closed reduction and cast application. Unstable ankle joints need internal fixation. When the closed reduction fails, patients are treated with ORIF and open reduction with external fixation. Charnley’s clamp is the most common method of external fixation used for ankle arthrodesis. External fixator systems or Ilizarov are used if ankle dislocations are associated with compound fractures.

Here, we present a case of a 27-year-old male patient who fell from a height of 4 feet on an uneven surface eight months back and was treated by slab application for one month, following which his symptoms subsided, but later noticed an equinus deformity and came to our hospital.

## Case presentation

A 27-year-old male patient came to our hospital with complaints of plantar flexion deformity and restriction of movement at the right ankle for seven months. The patient was well eight months back when he had a history of fall from height, following which he had severe pain and swelling in his right ankle, for which he was taken to the local hospital where his ankle was immobilized with the below knee slab and advised to follow up after one month.

After one month, when the patient went for a follow-up, the slab was removed, and it was seen that the patient's ankle was fixed in plantar flexion. An imaging study was done, and the patient was advised of surgical management. The patient declined the surgery as he was no longer experiencing pain. He noticed that his foot was still in plantar flexion, but he carried on his daily day-to-day activities by limping. As there was no improvement in deformity, the patient came to the outpatient department to get definitive management done, for which the patient was admitted.

**Figure 1:**
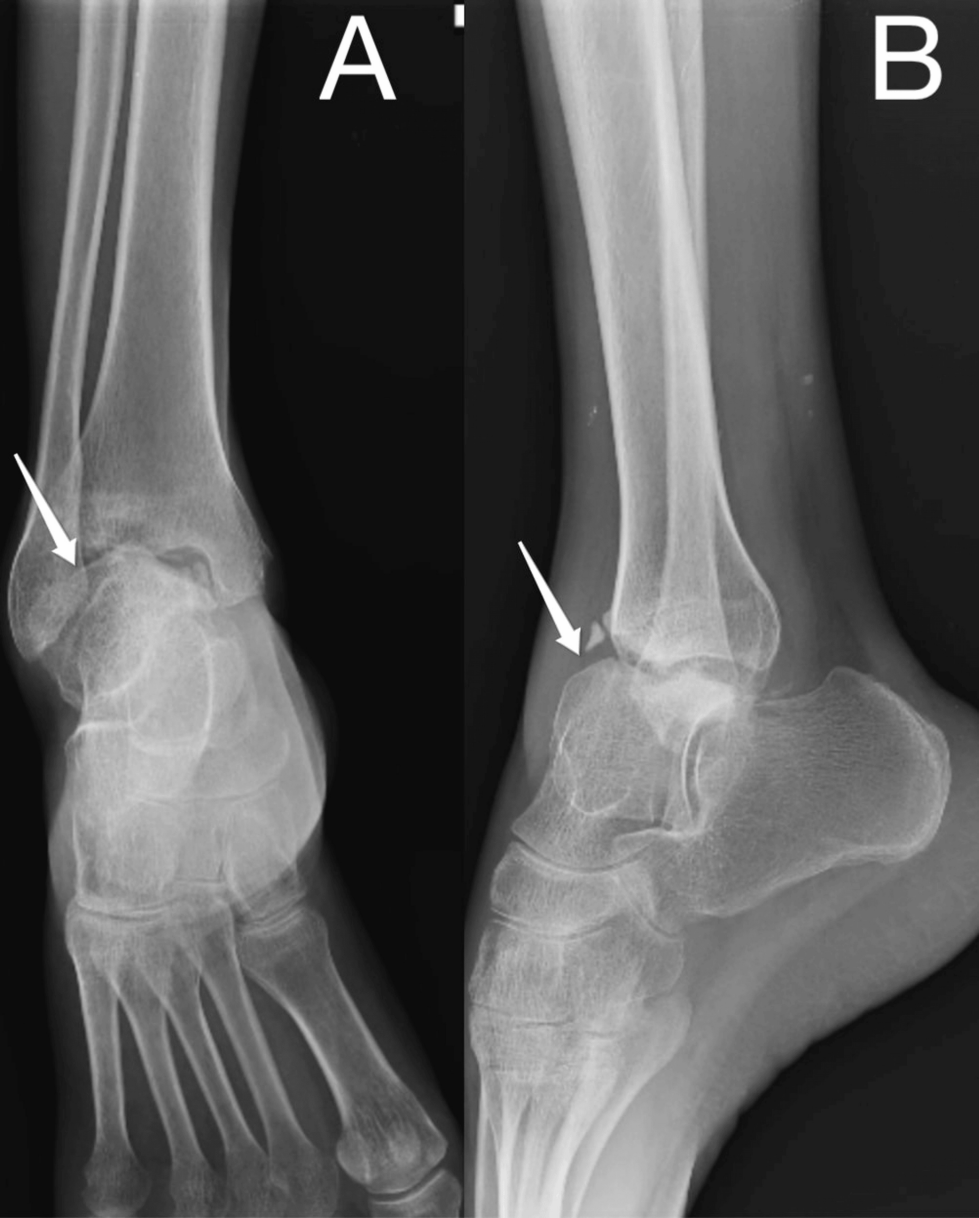
Preoperative radiograph of the right ankle (A) anteroposterior view and (B) lateral view. The white arrows show the subluxation of the talus

A detailed examination was done, and it was found that the right ankle was fixed in 20° plantar flexion with further flexion from 20° to 30°. An X-ray of the right ankle, anteroposterior and lateral views, was done, which showed displacement of the talus bone from the ankle mortise and rotation laterally. Along with it, a chip fracture from the anterior part of the tibia was seen. It also showed sclerosis of the margins of the talus and calcaneum with few osteophytes in the ankle and subtalar joint (Figure 1).

**Figure 2:**
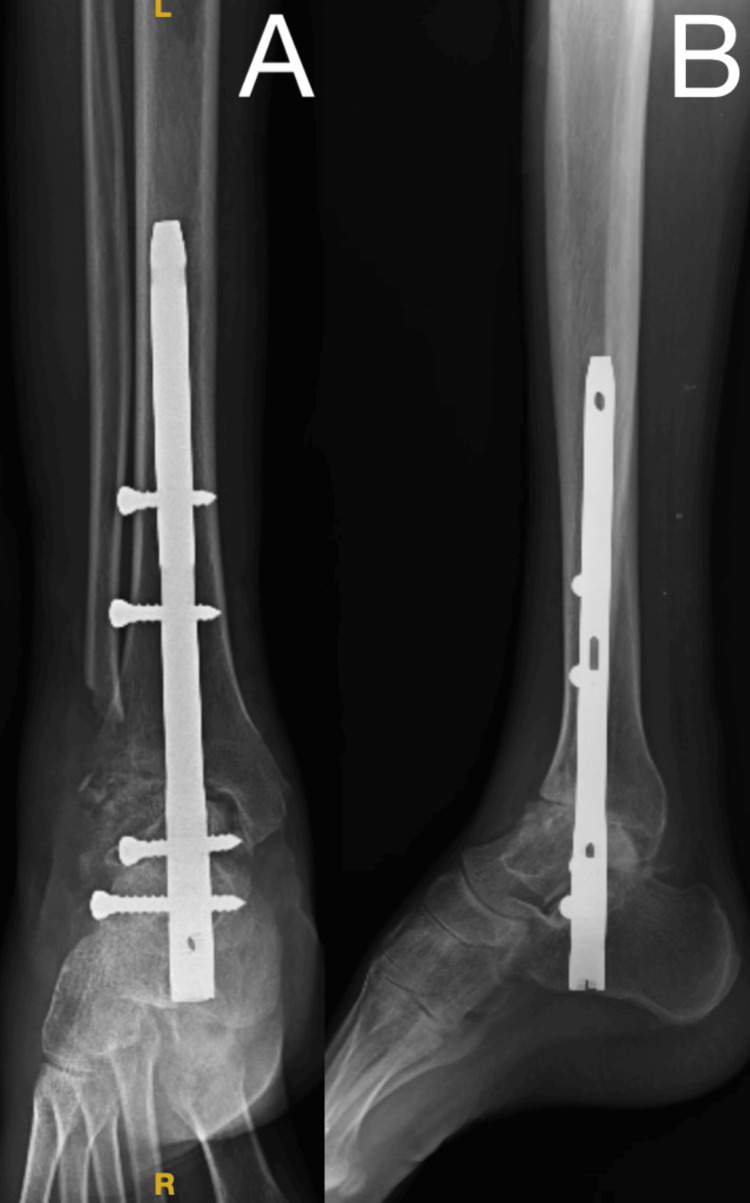
Postoperative radiograph of the right ankle (A) anteroposterior view and (B) lateral view

The patient underwent a procedure with a lateral approach. An 8 cm incision was made on the distal aspect of the right leg, starting 4 cm above the lateral malleolus and extending towards the base of the fourth metatarsal. Additionally, the distal 4 cm of the fibula was removed, and all fibrotic tissue and cartilage across the ankle joint were excised. The joint was reduced under the C-arm, and the bone grafting, which was harvested from the distal end fibula, was prepared and inserted over the joint. A TTC nail was inserted through the plantar aspect of the foot through the calcaneum and talus to the tibia, followed by proximal and distal locking. A postoperative radiograph showed a reduced gap in the ankle and subtalar joint and osteotomy of the distal end of the fibula and TTC nail with two proximal and two distal screws in situ (Figure 2). The stability of the ankle joint was also confirmed clinically.

The patient was allowed partial weight-bearing mobilization with the use of crutches for one week, followed by full weight-bearing mobilization. At the six-month follow-up, the patient was pain-free, his foot is in plantigrade, and he is able to ambulate without difficulty. The preoperative American Foot and Ankle Society score was 49%, with a significant improvement to 81% in six months. 

## Discussion

Treating complex fractures of the distal tibia, ankle, and talus with soft tissue damage, bone loss, and non-reconstructable joints for which the optimal timing for reduction and fixation has been missed is challenging [5]. In such cases, primary arthrodesis might be a treatment option. TTC fusion is a surgical procedure that aims to restrict movement of the TTC joint and relieve pain caused by ankle motion. The benefits of intramedullary nailing compared with other fusion methods include more load sharing during healing and less soft tissue injury, potentially leading to earlier weight-bearing [6]. Recently, TTC intramedullary nailing has been proposed as an alternative to ORIF for the first-line treatment of unstable joints with compromised soft tissue envelope that theoretically could avoid some things but not all short-term complications. The TTC nail offers a significant advantage in this scenario as we did with the patient who had long ignored deformities before considering this. It can reduce and stabilize the dislocated joint and begin early treatment or provide protection in case of soft tissue damage. Thanks to the internal fixation provided by the TTC nail, a more stable joint was created compared to traditional external fixation methods. The primary treatment for frail patients with severe bone loss who are contraindicated from ORIF or an alternative to amputation has been described as TTC stabilization by a retrograde intramedullary nail [5].

Therefore, on this advanced fixation method, the decision has to be made using this measure against these potential risks, and an individual approach must be issued according to the characteristics of the dislocation and the patient's overall condition.

## Conclusions

The utilization of a TTC nail, particularly in the context of long-standing ankle dislocations, signifies a significant advancement in addressing complex and long-neglected orthopedic injuries. This particular case serves as a compelling illustration of the remarkable stability achieved through internal fixation with the TTC nail, as well as the consequential functional recovery experienced in the face of challenging scenarios. There is a clear need for additional comprehensive studies and the compilation of further case reports to establish precise indications and refined techniques for the application of TTC nails in similar cases.
